# The emotion storyboard: A method to examine social judgments of emotion

**DOI:** 10.1371/journal.pone.0249294

**Published:** 2021-04-02

**Authors:** Kaitlin McCormick-Huhn, Stephanie A. Shields

**Affiliations:** 1 William S. Boyd School of Law, University of Nevada, Las Vegas, Las Vegas, Nevada, United States of America; 2 Department of Psychology, The Pennsylvania State University, University Park, Pennsylvania, United States of America; Victoria University of Wellington, NEW ZEALAND

## Abstract

As perceivers, we need to understand context to make social judgments about emotion, such as judging whether emotion is appropriate. We propose a graphic novel-like method, the emotion storyboard, for use in research on social judgments of emotion. Across two studies, participants were randomly assigned to read emotion storyboards or written vignettes to compare the efficacy of the emotion storyboard to that of vignettes in studies on social judgments of emotion. In Study 1, undergraduates (*N* = 194) answered comprehension questions and rated story clarity and immersion. Participants also made social judgments of emotion by rating main character emotion control and appropriateness of intensity. To further compare the efficacy of the methods, in Study 2, Amazon Mechanical Turk workers (*N* = 213) answered comprehension questions while response times were recorded, rated clarity, answered a race manipulation check, and rated main character emotion type appropriateness. Overall, emotion storyboards resulted in greater clarity ratings, greater race manipulation check accuracy, and in some instances, enhanced comprehension and comprehension response times relative to vignettes. In emotion storyboards, main character emotion was rated more controlled and more appropriate in intensity, but not different in emotion type appropriateness, than in vignettes. Overall, the method offers a new method of examining social elements of emotion that enhances comprehension and maximizes experimental efficiency.

## Introduction

Emotion is social. Emotion is often created in response to social elicitors, affected and regulated by others, and understood by the self through others’ reactions. Therefore, as perceivers, we need to understand the person’s context (i.e., the particular social setting) and the other people involved in the person’s situation to understand and make judgments about their emotion. In experimental work, vignettes are commonly used to assess social dimensions of hypothetical others’ emotion. As an alternative, we propose a new graphic novel-like method, the emotion storyboard, to assess social judgments of emotion in context while enhancing comprehension of content. Although cartoons are commonly used to depict emotion in psychological studies with children [1] and graphic novel formats are used to maximize comprehension in other disciplines [2], to the best of our knowledge, the graphic novel-like format is new in the psychological study of adult emotion. In the present research, we compare the effectiveness of emotion storyboards and vignettes as methods for examining social evaluations of emotion in context.

### Emotion is social

In recent years there has been a move from conceptualizing emotions as internal, private states to a perspective that recognizes emotion as social [3–6], in that emotion is affected by context and by the actions and reactions of other social actors within the context. Contextual cues affect both perceived type and intensity of emotion in oneself [7] and in others [8]. For instance, the happiness and pride of a face expressing happiness were rated as greater in the context of achieving a high exam grade than an average exam grade [9]. Simply the presence of other faces expressing emotion while looking at a target face influenced ultimate judgments of a target face’s emotion [10], even when the other faces were presented subliminally [11]. The study of emotion, therefore, requires methods that include social contextual information. We propose the emotion storyboard, by combining illustrations and text, may be well-positioned to examine emotion research questions about emotion that are affected by social context.

### Measuring emotion as social and social judgments of emotion

A variety of methods can be used to examine emotion as a social process, including face and full body photographs and morphs, audio, vignettes, vignettes with photographs, videos, and innovative methods, such as the Amsterdam Dynamic Facial Expression Set [12]. Different types of emotion-relevant questions, however, require different methods. For example, to depict emotion in context over an extended time-course, researchers often rely on vignettes or videos [13].

Vignettes are written descriptions of hypothetical events. Vignettes have long been used to examine social judgments of emotion. Social judgments of emotion include people’s perceptions, beliefs, stereotypes, and attitudes about hypothetical third-party others [14–16]. Vignettes can be particularly effective for examining social judgments of emotion because their hypothetical nature and third-party focus allow for depersonalized and unthreatening responding [15]. Additionally, vignettes can provide social contextual information that affects evaluations, such as whether emotion is under control and authentic [17], whether emotion is appropriate and typical [18], and the status of the person experiencing the emotion and their likely action tendencies [19]. Vignettes are also used to induce emotion or to stimulate people’s predictions of how they might act in a given situation. There are, of course, limitations to using vignettes, such as disconnection from social experiences in emotion induction and detachment from real-world consequences in hypothetical decision-making situations [20, 21]. In the present studies, we focused on the first type of vignette method: using vignettes to examine people’s social judgments about emotion.

### Why a graphic novel approach?

In the present studies we were most interested in comparing vignettes used for the perception and social evaluation of third-party others’ emotion in context with our proposed graphic novel-like method, the emotion storyboard. In graphic novel formats, illustrations and text are combined to convey stories. Our method, the emotion storyboard, uses a graphic novel-like format to visually represent emotion in context. In psychology, cartoons have long been used to measure emotion recognition and understanding in typically developing children [1, 22, 23] and children with autism spectrum disorders and other emotion-processing difficulties [24]. Cartoons have also been used to examine Theory of Mind reasoning [25] and to examine emotion cross-culturally [26]. To our knowledge, graphic novel-like formats using illustrations and text to create story plots have not been used as an alternative to vignettes to examine adult emotion.

The graphic novel has gained both popularity and legitimacy in public discourse [27] and is used in other academic fields for research and teaching [28]. The American Medical Association’s *Journal of Ethics*, for example, devoted a special issue to the study of graphic medicine (i.e., use of visual narratives; [29]). Graphic novel methods have been used to teach concepts in medicine [28], business [30], history [31], literacy [32], and secondary school [33]. Studies in these domains have demonstrated that graphic novels and illustrated content resulted in greater recall [30], enhanced test performance and reading behavior [34], greater reading motivation [2], and comparable decision-making while requiring less reading time [35] than text-only formats. Overall, graphic novel formats have resulted in greater memory of content, greater ease of processing, and greater motivation to engage with content, than text alone. Findings from other disciplines suggest that emotion storyboards could enhance comprehension of content relative to written vignettes in research on social judgments of emotion.

### A new method for obtaining judgments about others’ emotion: The emotion storyboard

In the present studies, we examined the relative effectiveness of emotion storyboards and vignettes for study manipulations of social emotion. We also conducted exploratory analyses for questions of interest that we did not have specific hypotheses for. In Study 1, we investigated whether the emotion storyboard method could convey information about social context comparable to the vignette method, while enhancing comprehension of information. We hypothesized a difference in effectiveness of the two methods on comprehension, clarity, and immersion. Our exploratory analyses in Study 1 examined whether the two methods yielded differences in social judgments of emotion, specifically character emotional control and appropriateness of emotional intensity.

In addition, we examined two mechanisms that could possibly explain greater comprehension for the emotion storyboard than vignette. First, because graphic novels have yielded greater ease of processing than text formats [35], we reasoned this could be due to the graphic presentation being clearer and less confusing than text. If so, greater clarity may explain enhanced comprehension. Second, based on findings of greater reported motivation to read graphic novels than text [30], we reasoned people might experience greater feelings of immersion or absorption in the graphic novel reading process. If so, greater immersion could also explain enhanced comprehension. Therefore, we predicted that participants would pass comprehension checks at a higher rate and rate the stories as clearer and more immersive in emotion storyboard than vignette conditions. We also predicted that clarity and immersion would mediate the relationship between format and comprehension. Finally, we explored how the emotion storyboard method would fare relative to the vignette method for evaluative judgments that are affected by social context, namely, perceptions of emotion as under control [17] and emotional intensity as appropriate [18].

In Study 2, we examined whether emotion storyboards may offer a way to maximize accuracy relative to vignettes for identifying social group memberships (e.g., race; socioeconomic status; gender) in studies about social emotion judgments. In vignettes it can be difficult to represent social group memberships without being too subtle (e.g., using names stereotypical of particular social groups). To test this possibility, we examined participants’ accuracy on a race manipulation check in emotion storyboards compared to only subtle name manipulations in vignettes. In Study 2, we again examined comprehension and clarity. We also explored whether emotion storyboards resulted in quicker comprehension question response time and lower overall story reading time compared to vignettes. Additionally, in Study 2 we conducted an exploratory test of the relative effectiveness of the two methods for evaluative judgments of emotion other than emotion expression. Specifically, in line with feeling rules [36], we investigated evaluations of the appropriateness of the emotion type for the given situation [37]. Pilot studies as well as Study 1 and Study 2 were between-subjects designs.

## Materials

Emotion storyboards and vignettes depicted a main character experiencing anger in the workplace, an emotion-relevant situation that has been the topic of studies on social judgments of emotion [19, 38]. Our goal was to compare a narrative vignette of the type commonly used to describe a person’s emotional experience [e.g., 17, 19] to the emotion storyboard. We developed a written vignette by describing a character becoming angry due to a situation at work, and then worked with a graphic artist to translate the written vignette into panels for the emotion storyboard version.

A graphic artist developed characters for the emotion storyboards and held constant gendered facial features (e.g., brow ridges) that are confounded with emotion expression type [39].

**Fig 1:**
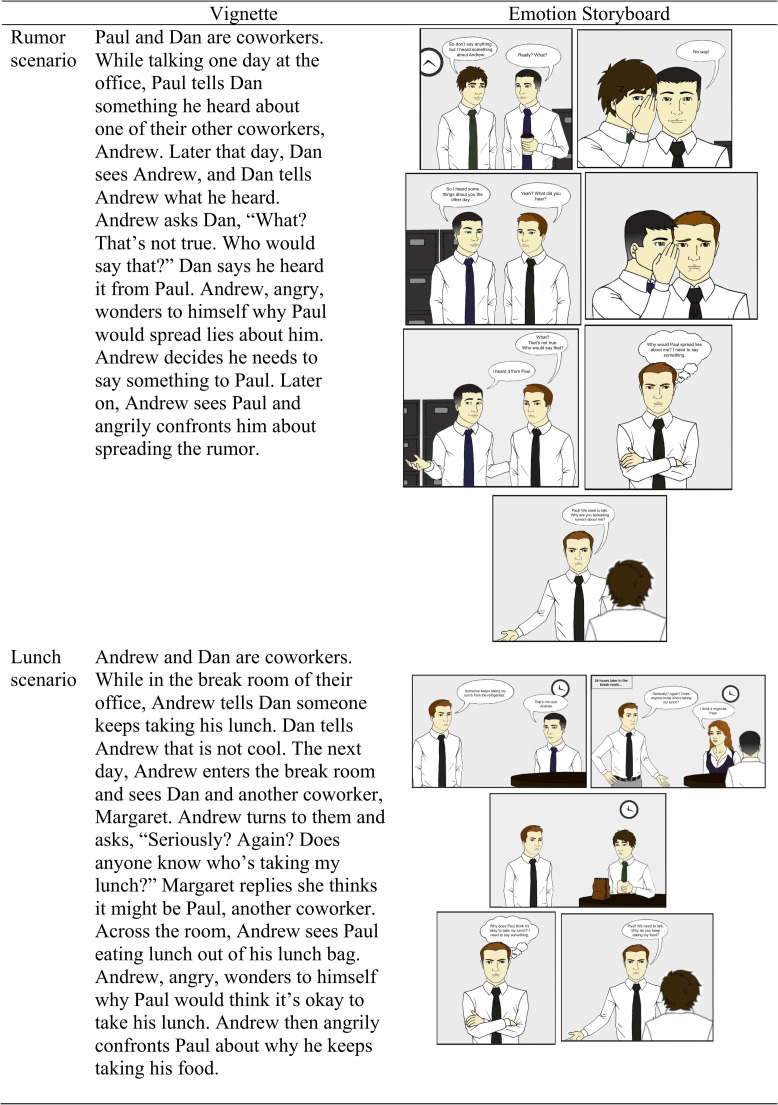
Study 1 vignette and emotion storyboard materials (illustrations by Michael Przybys).

For Study 1, each method was compared across two scenarios that involved a character experiencing workplace anger to ensure that participants’ judgments were not specific to features of the particular social situation. To select our two scenarios, we drew on reports of two surveys conducted with employees in business workplaces [40, 41] and selected three situations from the reports to pilot: taking credit for a coworker’s work, spreading a rumor about a coworker, and stealing a coworker’s lunch. Study 1 materials depicted a White man character (see Fig 1).

**Fig 2:**
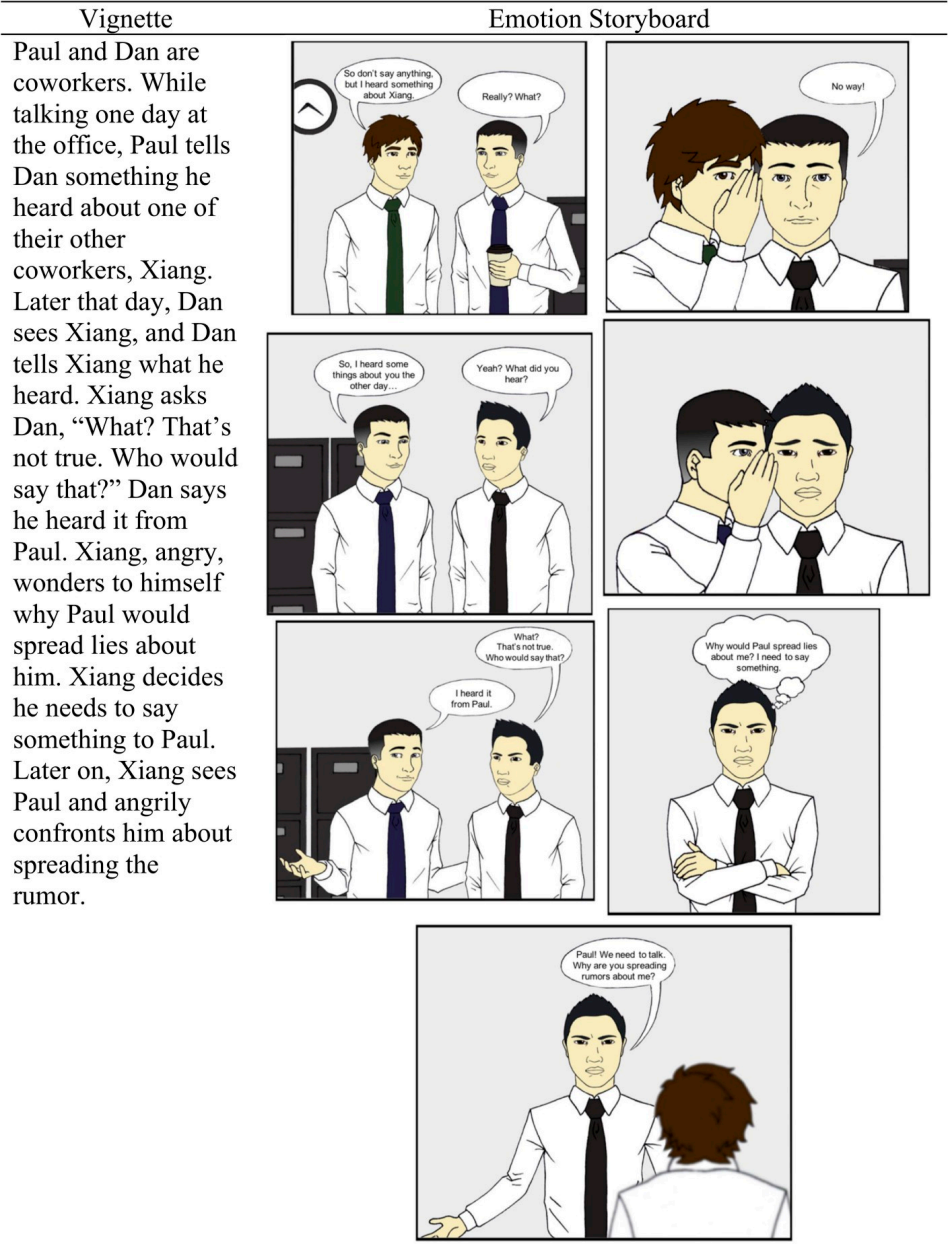
Study 2 additional vignette and emotion storyboard materials (illustrations by Michael Przybys).

For Study 2, we used the rumor vignette and emotion storyboard with the White man character from Study 1. We also used that same vignette and emotion storyboard with an Asian man character. To manipulate race in the vignette, we used Anglo and Chinese names, Andrew and Xiang, respectively, that were used previously in vignette research [42]. For the emotion storyboard, the White man character was modified to have Asian features, while keeping other properties of the face (e.g., brow ridges; eyebrow position) constant [39] (see Fig 2).

## Pilot studies

Four pilot studies were conducted to test equivalence of scenarios, accurate perception of main character’s gender and race, and type and intensity of emotion expressed by the main character in the final panel of the storyboard.

### Scenario pilot study

We first used a between-subjects design to pilot three scenarios (*N* = 247) to examine perceptions of how justified someone would be to feel angry in the various situations. Participants were 247 university undergraduates (125 women, 121 men, and 1 transgender person; ages 18–49, *M* = 19.71, *SD* = 3.72). The racial/ethnic composition of participants in the sample was predominantly White (77.3%; Asian/Asian-American, 6.9%; Black, 4.5%; multiracial, 3.6%; Latinx, 3.2%; Middle Eastern, 1.6%; Native American/Alaskan, 1.2%; Native Hawaiian or Pacific Islander, 0.4%; Southeast Asian, 0.4%; other, 0.9%). For the present study, we selected two scenarios that were rated equivalently on justification of the main character’s anger, (*t*(165) = 1.12, *p* = .266, *d* = 0.17, 95% CI [-0.13, 0.48]), on a scale of 1 (Not justified at all) to 7 (Very justified): a character at work learning that a rumor had been spread about them (*M* = 5.28, *SD* = 1.39), and a character discovering that a coworker was stealing their lunch from the break room (*M* = 5.02, *SD* = 1.59).

### Character gender and race in emotion storyboard pilot studies

Next, we conducted pilot studies to ensure gender and race of the main character in the emotion storyboards were accurately identified by at least 80% of the sample. For Study 1, participants were university undergraduates (*N* = 17; 9 women, 8 men; ages 18–25, *M* = 20.18, *SD* = 1.67). The racial/ethnic composition of participants in the sample was predominantly White (64.7%; Asian/Asian-American, 11.8%; Black, 11.8%; Latinx, 5.9%; Southeast Asian, 5.9%). Participants provided open-ended responses that identified the main character as White (94%) and man (100%).

For Study 2, a sample of 18 university undergraduates were recruited for a pilot study (8 women, 9 men, and 1 other; ages 19–39, *M* = 20.78, *SD* = 4.67). The racial/ethnic composition of participants in the sample was predominantly White (61.1%; Black, 22.2%; Asian/Asian-American, 11.1%; Latinx, 5.6%). Participants provided open-ended responses that identified the main character as Asian (83%) and man (100%).

### Anger expression in emotion storyboard pilot study

University undergraduates in a psychology class participated in a pilot study, with a between-subjects design (*N* = 25; demographics not collected). Participants rated only the final panel, showing the anger expression of the character without text. The emotional intensity of a number of emotions was rated on a scale of Not at all (1) to Very (7). Piloting was conducted to ensure that the character was rated as more than moderately angry (indicated by a rating greater than 4 on a scale of Not angry at all (1) to Very angry (7); metric selected to account for perceptual overlap of facial emotion expressions when presented in a decontextualized manner [43]). For Study 1, participants who rated the White man main character (*N* = 7) perceived him as a good deal angry (*M* = 4.86, *SD* = 0.38), as somewhat disgusted (*M* = 2.86, *SD* = 1.46), somewhat sad (*M* = 2.43, *SD* = 1.13), slightly surprised (*M* = 2.14, *SD* = 1.07), and close to not at all afraid (*M* = 1.43, *SD* = 0.79) and to not at all happy (*M* = 1.29, *SD* = 0.49). For Study 2, participants who rated the Asian man main character (*N* = 5) perceived him as a good deal angry (*M* = 4.80, *SD* = 1.10), as moderately disgusted (*M* = 3.80, *SD* = 0.45), somewhat surprised (*M* = 3.60, *SD* = 1.67), and close to not at all sad (*M* = 1.40, *SD* = 0.55), to not at all afraid (*M* = 1.40, *SD* = 0.55) and to not at all happy (*M* = 1.20, *SD* = 0.45).

## Methods: Study 1

### Design

The design of the study was a 2 (format: emotion storyboard vs. vignette) X 2 (scenario: rumor vs. lunch stealing) between-subjects randomized design.

### Participants

An a priori power analysis for between-subject group comparisons in G*Power [44] revealed a sample size of 171 people was needed to detect a moderate effect size (*d* = 0.50) at .90 power. The Institutional Review Board (IRB) at The Pennsylvania State University’s Office of Research Protections approved the study. Psychology undergraduates in the United States participated online, remotely through a university-hosted site for course credit. As approved by the IRB, participants read a consent form before the study began that included a closing message that stated, “Your participation implies your voluntary consent.” Due to the online nature of the study, participants did not sign the consent form but instead indicated their consent by advancing to the next page. All participants chose to continue to the online study, indicating their consent. Pre-participation instructions asked participants not to complete the study on a phone to ensure participants viewed stimuli similar in size. We recruited 203 participants to account for potential exclusions. The final sample had 194 participants (96 women and 98 men; ages 18–30, *M* = 19.11, *SD* = 1.53) after exclusions (6 removed for completing the study on a phone, 3 more removed for failing an attention check). The racial/ethnic composition of participants in the sample was predominantly White (71.7%; Asian/Asian-American, 12.4%; Latinx, 5.7%; multiracial, 4.1%; Black, 3.6%; Middle Eastern, 1.5%; Native Hawaiian or Pacific Islander, 0.5%; Native American/Alaskan, 0.5%; other, 0.5%).

### Statistical reporting

For all data analyses, significance was determined using an alpha of *p* < .05. All analyses for pilot studies, Study 1, and Study 2, were conducted using SPSS Statistics [45].

### Procedure

Participants completed the study online and were told the purpose of the study was to examine people’s thoughts about social situations in the workplace. Participants were told they would read a brief story about a main character named Andrew. The design of the study was between-subjects, in which participants read one of two scenarios, in either emotion storyboard or vignette form (see Fig 1). In the rumor scenario, participants read about coworkers Paul, Dan, and the main character, Andrew. Paul tells Dan a rumor about Andrew, Dan then tells Andrew the rumor, and Andrew later confronts Paul. In the lunch stealing scenario, participants read about coworkers Dan, Margaret, Paul, and the main character, Andrew. Andrew expresses to Dan, and later to Dan and Margaret that someone keeps stealing his lunch. Margaret suggests it may be Paul and Andrew confronts Paul. For the emotion storyboard conditions, instructions also included an illustration of Andrew. After reading a scenario, participants answered comprehension and emotion manipulation check questions, rated story clarity and immersion, and rated character emotional control and appropriateness of emotional intensity.

### Measures

Participants responded to the following items (see S1 Appendix; scales created for this study unless noted).

### Manipulation checks

#### Emotion type/intensity

To ensure that the main character was judged as experiencing anger across format and scenario, we measured anger as a manipulation check. We also measured additional emotions (disgust, sadness, surprise, and fear) that could be relevant for the character in the scenarios. Five single-items assessed perceptions of how angry, sad, afraid, surprised, and disgusted the main character was when saying something to Paul (the wrongdoer) on 7-point Likert scales ranging from 1 (Not at all) to 7 (Very).

### Dependent measures

#### Comprehension

Comprehension was measured with two items, “Why was the main character angry?”; “Who did the main character confront?” (*r*_*s*_ (192) = 0.29, *p* < .001). Each item had four possible multiple-choice responses, later coded as correct or incorrect. Comprehension scores represented the proportion of correct answers.

##### Clarity

Clarity was measured with four items on 7-point Likert scales ranging from 1 (Not at all) to 7 (Very), “How easy was it to follow what was happening between the characters in the story?”; “How clear was the story you read?”; “How confusing was the story you read?” (reverse-coded, R); “How difficult was it to follow what emotions the characters in the story were feeling?” (R) (Cronbach’s α = .86).

#### Immersion

Immersion was measured with four items on 7-point Likert scales ranging from 1 (Not at all) to 7 (Very), “How absorbed in the story were you?”; “How interesting was the story you read?”; “How immersed in the story were you?”; “To what extent did you identify with any of the characters in the story?” (Cronbach’s α = .86).

### Exploratory measures

#### Emotional control

Emotional control was measured with an existing measure [17] of three items on 7-point Likert scales ranging from 1 (Not at all) to 7 (Very much), “How much self-control did the main character demonstrate?”; “How much were the main character’s feelings kept ‘in check’?”; “How much composure did the main character demonstrate?” (Cronbach’s α = .86).

#### Appropriateness of emotional intensity

Appropriateness of emotional intensity was measured with an existing measure [37] of five items on 7-point Likert scales ranging from 1 (Strongly disagree) to 7 (Strongly agree), “The emotions shown by the main character were too extreme (R)”; “Most people would not have been so emotional at certain points as the main character was.: (R); “The main character was too emotional.” (R); “I think the main character was emotionally out of control” (R); “I think that the main character had too much emotion for clear thinking” (R) (Cronbach’s α = .89).

## Results: Study 1

For the manipulation checks, dependent measures, and exploratory measures, we conducted 2 (format: emotion storyboard vs. vignette) X 2 (scenario: rumor vs. lunch stealing) between-subjects ANOVAs. To examine whether clarity or immersion mediated the effect of format (coded: 0 = vignette, 1 = emotion storyboard) on comprehension, we conducted two mediation analyses using the bootstrapping procedure, with 5,000 bootstrap samples and 95% confidence intervals, of the PROCESS macro for SPSS [46]. Correlations among variables are shown in Table 1. Means and standard deviations by format are listed in Table 2.

**Table 1:** Study 1 correlations between variables (across conditions).

	Comprehension	Clarity	Immersion	Emotional Control	Appropriateness of Emotional Intensity
Comprehension	1.00	.16*	.05	.06	.04
Clarity	.16*	1.00	.16*	.22**	.19*
Immersion	.05	.16*	1.00	.19**	.08
Emotional Control	.06	.22**	.19**	1.00	.50**
Appropriateness of Emotional Intensity	.04	.19*	.08	.50**	1.00

Note

* *p* < .05

** *p* < .01.

**Table 2:** Study 1 means (standard deviations) by format.

Measure (range of possible scores)	Comprehension (0.00–1.00)	Clarity (1–7)	Immersion (1–7)	Emotional Control (1–7)	Appropriateness of Emotional Intensity (1–7)
Emotion Storyboard	0.96 (0.13)^a^	6.07 (0.95)^a^	3.18 (1.27)	3.85 (1.18)^a^	5.29 (1.17)^a^
Vignette	0.90 (0.23)^b^	5.49 (1.31)^b^	3.26 (1.35)	3.14 (1.11)^b^	4.58 (1.19)^b^
Effect size of difference (*η*_*p*_^*2*^*)*	.02	.06	.001	.09	.08

Note: Different subscripts indicate the two format conditions significantly differed from one another on the variables

of interest (by at least *p* < .05).

### Manipulation checks

#### Emotion type/intensity

For anger intensity, differences based on format were nonsignificant, *p* = .240, *η*_*p*_^*2*^ = .01, 95% CI [0.00 to 0.05] (emotion storyboards *M* = 5.66, *SD* = 1.26; vignettes *M* = 5.87, *SD* = 1.22). Differences based on scenario were nonsignificant (*p* = .400, *η*_*p*_^*2*^ = .004, 95% CI [0.00 to 0.04]). Differences based on the interaction between format and scenario were also nonsignificant (*p* = .259, *η*_*p*_^*2*^ = .01, 95% CI [0.00 to 0.05]).

For fear, disgust, surprise, and sadness, differences based on format were nonsignificant (see S2 Appendix for further reporting of manipulation check results for emotions other than anger). For fear, disgust, and surprise, differences based on the interaction of format and scenario were also nonsignificant. Perceptions of the main character’s sadness differed based on the interaction between format and scenario (*F*(1, 190) = 4.16, *p* = .043, *η*_*p*_^*2*^ = .02, 95% CI [0.00 to 0.08]). Simple effects testing of the interaction revealed there was an effect (*F*(1, 190) = 13.93, *p* < .001, *η*_*p*_^*2*^ = .07, 95% CI [0.02 to 0.15]) in the emotion storyboard format of the main character being perceived as sadder in the rumor situation (*M* = 3.42, *SD* = 1.68) than in the lunch stealing situation (*M* = 2.23, *SD* = 1.32).

### Dependent measures

#### Comprehension

There was an effect of format on comprehension, *F*(1, 190) = 4.38, *p* = .038, *η*_*p*_^*2*^ = .02, 95% CI [0.00 to 0.08], such that participants’ comprehension was greater in emotion storyboard than vignette conditions (see Table 2 for means and standard deviations). Differences based on scenario were nonsignificant (*p* = .225, *η*_*p*_^*2*^ = .01, 95% CI [0.00 to 0.05]). Differences based on the interaction between format and scenario were also nonsignificant (*p* = .324, *η*_*p*_^*2*^ = .01, 95% CI [0.00 to 0.04]).

#### Clarity

There was an effect of format on clarity, *F*(1, 190) = 12.80, *p* < .001, *η*_*p*_^*2*^ = .06, 95% CI [0.01 to 0.14], such that participants rated the stories as clearer in emotion storyboard than vignette conditions (see Table 2). Differences based on scenario were nonsignificant (*p* = .637, *η*_*p*_^*2*^ = .001, 95% CI [0.00 to 0.03]). Differences based on the interaction between format and scenario were also nonsignificant (*p* = .848, *η*_*p*_^*2*^ = .00, 95% CI [0.00 to 0.01]).

#### Immersion

Differences in immersion based on format were nonsignificant (*p* = .607, *η*_*p*_^*2*^ = .001, 95% CI [0.00 to 0.03]; see Table 2). Differences in immersion based on scenario were nonsignificant, (*p* = .069, *η*_*p*_^*2*^ = .02, 95% CI [0.00 to 0.07]). Differences based on the interaction between format and scenario were nonsignificant (*p* = .747, *η*_*p*_^*2*^ = .001, 95% CI [0.00 to 0.02]).

#### Mediation analyses

Neither clarity (*b* = -0.01, 95% bootstrapped CI: [-0.03, 0.00]), nor immersion (*b* = 0.001, 95% bootstrapped CI: [-0.002, 0.01]), had a significant indirect effect on comprehension.

### Exploratory variables

#### Emotional control

There was an effect of format on emotional control, *F*(1, 190) = 17.87, *p* < .001, *η*_*p*_^*2*^ = .09, 95% CI [0.02 to 0.17], such that the main character was rated as higher in emotional control in emotion storyboard than vignette conditions (see Table 2). Differences based on scenario were nonsignificant (*p* = .229, *η*_*p*_^*2*^ = .01, 95% CI [0.00 to 0.05]). Differences based on the interaction between format and scenario were also nonsignificant (*p* = .761, *η*_*p*_^*2*^ = .001, 95% CI [0.00 to 0.02]).

#### Appropriateness of emotional intensity

There was an effect of format on appropriateness of emotional intensity, *F*(1, 190) = 16.83, *p* < .001, *η*_*p*_^*2*^ = .08, 95% CI [0.02 to 0.16], such that the main character’s emotional intensity was rated more appropriate in emotion storyboard than vignette conditions (see Table 2). There was also an effect of scenario on appropriateness of emotional intensity, *F*(1, 190) = 12.93, *p* < .001, *η*_*p*_^*2*^ = .06, 95% CI [0.01 to 0.14], such that the character’s emotional intensity was rated more appropriate in the rumor situation (*M* = 5.23, *SD* = 1.04) than lunch stealing situation (*M* = 4.60, *SD* = 1.33). Differences based on the interaction between format and scenario were nonsignificant (*p* = .619, *η*_*p*_^*2*^ = .001, 95% CI [0.00 to 0.03]).

## Discussion: Study 1

Manipulation check findings suggest the main character’s anger when confronting the wrongdoer was rated equivalently across conditions. However, the unexpected interaction of the main character in the emotion storyboard being rated as sadder in the rumor than lunch-stealing scenario may suggest that visual depictions from previous panels in the emotion storyboard affected the judgment of the main character’s emotion when he was confronting the wrongdoer. Alternatively, this interaction may have been a Type I error due to the number of tests conducted.

Study 1 findings suggest the emotion storyboard method enhances comprehension and clarity, but not immersion, of story content relative to vignettes. Contrary to predictions, neither clarity nor immersion mediated the effect of format on comprehension. Comprehension was, however, weakly correlated with clarity and not associated with other measures (see Table 1). It remains possible, given comprehension in both conditions was relatively high, that clarity may mediate the emotion storyboard’s effectiveness on comprehension if comprehension questions were more difficult. Therefore, in Study 2, we examined the relationship between comprehension and clarity again for the two methods with three comprehension questions. One of the comprehension questions used in Study 1 about confrontation may have been ambiguous in the emotion storyboard conditions, given that the main character confronts several characters about the situation that evoked their anger, whereas only one interaction is labeled as confrontation in the vignette. In Study 2, we replaced this question with a more neutrally-worded question assessing how the character discovered who the wrongdoer was. We also added a question asking how many total characters were portrayed in the story.

Findings that emotion storyboards were clearer than vignettes may suggest that emotion storyboards are easier to process for participants. To examine this possibility in Study 2 we also compared participants’ overall time spent reading the story in the two format conditions. To explore the potential efficiency of the emotion storyboard method, we also examined the average response times of participants to the comprehension questions.

Additionally, participants evaluated the main character as expressing emotion with more emotional control and more appropriate emotion intensity in emotion storyboard than vignette conditions, suggesting that the emotion storyboard method allows for needed experimental control in depictions of expressions of emotion. To explore whether the two methods differ in broader emotion judgments, not specifically about emotion expression, in Study 2 we also examined evaluations of the main character’s appropriateness of emotion type for the given situation.

In Study 2, we also compared the efficacy of two methods to convey information about a character’s social group membership because such information is often manipulated in research on social judgments about emotion. To compare the methods on this dimension, we examined whether experimental manipulations of race in emotion storyboard resulted in greater accuracy on a race manipulation check compared to subtle name manipulations in vignettes.

## Method: Study 2

We chose to use only the rumor scenario for Study 2 because it was the longer of the two scenarios from Study 1. We examined accuracy on a race manipulation check, comprehension, clarity, story reading time, average comprehension question response time, and appropriateness of emotion type experienced by the main character.

### Design

The design of the study was a 2 (format: emotion storyboard vs. vignette) X 2 (character race: Asian man vs. White man) between-subjects randomized design.

### Participants

An a priori power analysis for between-subject group comparisons in G*Power [44] revealed a sample size of 171 people needed to detect a moderate effect size (*d* = 0.50) at .90 power. The study was approved by the IRB at The Pennsylvania State University’s Office of Research Protections and involved the same process for consent as in Study 1. Workers on Amazon Mechanical Turk (MTurk) in the United States participated online. All participants chose to continue to the online study, indicating their consent. Pre-participation instructions asked participants not to complete the study on a phone to ensure participants viewed stimuli similar in size. We recruited 226 participants to account for potential exclusions. The final sample had 213 participants (96 women and 117 men; ages 18–68, *M* = 35.52, *SD* = 11.33) after exclusions (8 removed for completing the study on a phone, 5 more removed for failing an attention check). The racial/ethnic composition of participants in the sample was predominantly White (71.8%; Asian/Asian-American, 8.9%; Black, 8.9%; Latinx, 6.6%; multiracial, 0.9%; Middle Eastern, 0.5%; Native Hawaiian or Pacific Islander, 0.5%; Native American/Alaskan, 1.9%).

### Procedure

As in Study 1, the design was between subjects. Participants read either an emotion storyboard or a vignette, with either an Asian man (Xiang) or a White man (Andrew) as the main character (see Fig 2). For the Asian man vignette condition, participants read about a main character named Xiang. For the Asian man emotion storyboard condition, participants read about and viewed a depiction of an Asian man character referred to by the name Xiang. We selected Anglo and Chinese names, Andrew and Xiang, respectively, that were used previously in vignette research [42]. In Study 2, only the rumor scenario from Study 1 was used. Participants read about coworkers Paul, Dan, and the main character, Xiang/Andrew. Paul tells Dan a rumor about Xiang/Andrew, Dan then tells Xiang/Andrew the rumor, and Xiang/Andrew later confronts Paul. For the emotion storyboard conditions, instructions also included an illustration of Xiang or Andrew. Participants completed the study online through MTurk and the procedure was the same as in Study 1.

### Measures

A list of all items for Study 2 measures is available in S3 Appendix.

### Dependent measures

#### Race manipulation check

The accuracy of the race manipulation check was measured with one, open-ended item at the end of the study, “What do you think was the race/ethnicity of the main character who became angry?” Each response was coded as correct or incorrect. Responses that identified the correct racial group or an ethnicity within the correct racial group (e.g., Chinese) were coded as correct. Responses that were incorrect, unsure, or did not provide a race were coded as incorrect. There were two responses, one in an emotion storyboard condition and one in a vignette condition, for which coding was less clear. For the emotion storyboard response, the participant provided two groups, one of which was the correct racial group. For the vignette response, the participant labeled the White main character as “Not Hispanic.” We opted to code both responses as correct. The pattern of results was unchanged when these two responses were coded as correct or as incorrect. All free-text responses to this measure are available in the Study 2 dataset.

#### Comprehension

Comprehension was measured with three items, “Why was the main character angry?”; “How did the main character discover that it was Paul who spread a rumor about him?”; “How many characters were there in the story you read?” (Cronbach’s α = .63). Each item had four possible multiple-choice responses, later coded as correct or incorrect. Comprehension scores represented the proportion of correct answers.

### Clarity

Clarity was measured with Study 1 items (Cronbach’s α = .87).

### Exploratory measures

#### Reading time

Reading time was measured by calculating how long participants remained on the story page before clicking the arrow to advance the online survey.

#### Comprehension response time

Comprehension response time was measured by calculating the average time that participants remained on the question page before clicking the arrow to advance to the next question, across the three comprehension questions.

#### Appropriateness of emotion type

Appropriateness of the emotion type was measured with an existing measure [37] of four items on 7-point Likert scales ranging from 1 (Strongly disagree) to 7 (Strongly agree), “The main character’s emotions were exactly the kinds that were called for.”; “I think the types of emotions that the main character felt were normal.”; “The emotions displayed by the main character were wrong.” (R); “I would not have shown the types of emotions that the main character displayed.” (R) (Cronbach’s α = .80).

## Results: Study 2

We conducted 2 (format: emotion storyboard vs. vignette) 2 (character race: Asian man vs. White man) between-subjects ANOVAs for all measures. To examine whether clarity mediated the effect of format (coded: 0 = vignette, 1 = emotion storyboard) on comprehension, we conducted a mediation analysis using the bootstrapping procedure, with 5,000 bootstrap samples and 95% confidence intervals, of the PROCESS macro for SPSS [46].

For the exploratory measure of reading time, participants with reading times two standard deviations above the average reading time across condition were excluded from the analysis (all five participants excluded were in vignette condition; no reading times were two standard deviations below the mean). Data were not normally distributed for overall reading time as assessed by Shapiro-Wilk’s test (for White man main character vignette condition *p* = .045; for all other conditions *p*s < .001). Neither a log transformation nor a square root transformation resulted in conditions with normally distributed data. Therefore, we conducted the ANOVA without a transformation because ANOVAs can be relatively robust with deviations from normality [47].

For comprehension response time, participants with an average response time two standard deviations above the average across condition were excluded from the analysis (seven participants in vignette condition, one participant in the emotion storyboard condition; no average response times were two standard deviations below the mean). Data were not normally distributed for comprehension response time as assessed by Shapiro-Wilk’s test (*p*s < .001 in the four cells). Neither a log transformation nor a square root transformation resulted in conditions with normally distributed data. Thus, we conducted the ANOVA without a transformation.

For all measures, correlations among variables are shown in Table 3. Means and standard deviations by format are listed in Tables 4 and 5.

**Table 3:** Study 2 correlations (across conditions).

	Comprehension	Comprehension Response Time	Race Manipulation Check	Clarity	Overall Reading Time	Appropriateness of Emotion Type
Comprehension	1.00	-.09	.21**	.46**	-.01	.42**
Comprehension Response Time	-.09	1.00	-.04	-.10	.34**	-.14*
Race Manipulation Check	.21**	-.04	1.00	.13	-.07	.17*
Clarity	.46**	-.10	.13	1.00	-.09	.42**
Overall Reading Time	-.01	.34**	-.07	-.09	1.00	-.03
Appropriateness of Emotion Type	.42**	-.14*	.17*	.42**	-.03	1.00

Note:

* *p* < .05

** *p* < .01. Outliers on Comprehension Response Time and Overall Reading Time were excluded for this analysis.

**Table 4:** Study 2 means (standard deviations) by format.

Measure (range of possible scores)	Race Manipulation Check (0.00–1.00)	Comprehension (0.00–1.00)	Clarity (1–7)	Appropriateness of Emotion Type (1–7)
Emotion Storyboard	0.86 (0.35)^a^	0.91 (0.24)	6.11 (1.12)^a^	5.71 (1.15)
Vignette	0.73 (0.45)^b^	0.92 (0.18)	5.69 (1.40)^b^	5.51 (1.20)
Effect size of difference (*η*_*p*_^*2*^*)*	.03	.00	.03	.01

Note: Different subscripts indicate the two format conditions significantly differed from one another on the variables

of interest (by at least *p* < .05).

**Table 5:** Study 2 means (standard deviations) of reading and average comprehension response times in seconds by format.

Measure	Overall Reading Time	Comprehension Response Time
Emotion Storyboard	23.74 (15.83)	20.46 (9.42)^a^
Vignette	27.44 (18.58)	24.58 (15.94)^b^
Effect size of difference (*η*_*p*_^*2*^*)*	.01	.02

Note: Different subscripts indicate the two format conditions significantly differed from one another on the variables of interest (by at least *p* < .05).

### Dependent measures

#### Race manipulation check

Accuracy at identifying the main character’s race differed based on format, *F*(1, 209) = 5.99, *p* = .015, *η*_*p*_^*2*^ = .03, 95% CI [0.001 to 0.08], such that participants provided the correct race of the character at a higher rate in emotion storyboard than vignette conditions (see Table 4 for means and standard deviations). Differences based on main character race were nonsignificant (*p* = .368, *η*_*p*_^*2*^ = .004, 95% CI [0.00 to 0.04]). Differences based on the interaction between format and main character race were also nonsignificant (*p* = .153, *η*_*p*_^*2*^ = .01, 95% CI [0.00 to 0.05]).

#### Comprehension

Differences in comprehension based on format were nonsignificant, *F*(1, 209) = 0.07, *p* = .785, *η*_*p*_^*2*^ = .00, 95% CI [0.00 to 0.01] (see Table 4). Differences based on main character race were nonsignificant (*p* = .535, *η*_*p*_^*2*^ = .002, 95% CI [0.00 to 0.03]). Differences based on the interaction between format and main character race were also nonsignificant (*p* = .231, *η*_*p*_^*2*^ = .01, 95% CI [0.00 to 0.05]).

#### Clarity

Clarity differed based on format, *F*(1, 209) = 6.19, *p* = .014, *η*_*p*_^*2*^ = .03, 95% CI [0.001 to 0.09], such that participants rated the stories as clearer in emotion storyboard than vignette conditions (see Table 4). Clarity also differed based on main character race *F*(1, 209) = 6.71, *p* = .010, *η*_*p*_^*2*^ = .03, 95% CI [0.002 to 0.09], such that participants rated the stories as clearer when they read about an Asian man than a White man. Clarity differed based on the interaction between format and main character race as well, *F*(1, 209) = 4.56, *p* = .034, *η*_*p*_^*2*^ = .02, 95% CI [0.00 to 0.07]. Simple effects testing of the interaction revealed there was an effect (*F*(1, 209) = 11.11, *p* = .001, *η*_*p*_^*2*^ = .05, 95% CI [0.01 to 0.12]) in the vignette format of the stories being rated as clearer with the Asian man main character (*M* = 6.08, *SD* = 1.04) than with the White man main character (*M* = 5.28, *SD* = 1.61).

#### Mediation analyses

The indirect effect of format on comprehension with clarity as a mediator was significant (*b* = 0.03, 95% bootstrapped CI: [0.01, 0.06]).

### Exploratory measures

#### Reading time

Differences in reading time based on format were nonsignificant, *F*(1, 204) = 2.43, *p* = .121, *η*_*p*_^*2*^ = .01, 95% CI [0.00 to 0.06] (see Table 5 for means and standard deviations). Differences based on main character race were nonsignificant (*p* = .556, *η*_*p*_^*2*^ = .002, 95% CI [0.00 to 0.03]). Differences based on the interaction between format and main character race were also nonsignificant (*p* = .663, *η*_*p*_^*2*^ = .001, 95% CI [0.00 to 0.03]).

#### Comprehension response time

Average comprehension response time differed based on format *F*(1, 201) = 5.03, *p* = .026, *η*_*p*_^*2*^ = .02, 95% CI [0.00 to 0.08], such that participants responded to comprehension questions more quickly in emotion storyboard than vignette conditions (see Table 5). Differences based on main character race were nonsignificant (*p* = .454, *η*_*p*_^*2*^ = .003, 95% CI [0.00 to 0.03]). Differences based on the interaction between format and main character race were also nonsignificant (*p* = .424, *η*_*p*_^*2*^ = .003, 95% CI [0.00 to 0.04]).

#### Appropriateness of emotion type

Differences in appropriateness of emotion type based on format were nonsignificant, *F*(1, 209) = 1.38, *p* = .241, *η*_*p*_^*2*^ = .01, 95% CI [0.00 to 0.04] (see Table 4). Differences based on main character race were nonsignificant (*p* = .332, *η*_*p*_^*2*^ = .01, 95% CI [0.00 to 0.04]). Differences based on the interaction between format and main character race were also nonsignificant (*p* = .119, *η*_*p*_^*2*^ = .01, 95% CI [0.00 to 0.06]).

## Discussion: Study 2

Study 2 findings suggest the emotion storyboard method does enhance the accuracy of participants’ performance on a race manipulation check relative to the vignette method. And, as in Study 1, emotion storyboards were rated as higher in clarity than vignettes. In contrast to Study 1, differences in comprehension scores for story content in Study 2 were nonsignificant across method. However, clarity was moderately correlated with comprehension in Study 2 (see Table 3) and the indirect mediation of clarity for format on comprehension was significant, though weak in effect, in Study 2. Overall, high comprehension and clarity ratings suggest effects of clarity and format on comprehension may be more pronounced if story content was more complex and comprehension questions were still more difficult.

Unexpectedly, for clarity, an interaction and main effect of main character race emerged that suggest, especially for participants reading vignettes, that reading about an Asian character resulted in higher perceptions of clarity than reading about a White character did. This finding could be due to our predominantly White participants being especially attentive upon encountering a novel name. Alternatively, this interaction may have been a Type I error due to the number of tests conducted.

Across methods, reading time was equivalent for the story content, however, average comprehension question response time was faster for participants who read the story in emotion storyboard than in vignette. Such findings suggest the emotion storyboard method is able to maximize efficiency without damaging comprehension performance.

Additionally, judgments about the appropriateness of emotion type did not differ across methods. Patterns suggest that both the emotion storyboard and vignette adequately conveyed that the situation was one in which anger would be an appropriate response for the main character.

## Discussion: General

Overall, findings suggest the emotion storyboard is comparable to the vignette method, and has some advantages for assessing evaluations of third-party others’ emotion. Findings suggest emotion storyboards enhance clarity, and in some instances, comprehension and comprehension response time, relative to vignettes when conveying stories of the same complexity. Although effect sizes are small to medium for comprehension, comprehension response time, and clarity, and thus for some of the measures somewhat lower than our sample had adequate power to detect, even small gains on these dimensions could be meaningful for enhancing presentation to participants of information pertaining to social elements of emotion and social context. Findings are in line with effects found in studies comparing graphic novels to text in other disciplines [30, 35].

The emotion storyboard method also facilitated greater accuracy on a race manipulation check than the vignette method. On a practical level, using the emotion storyboard to manipulate race would limit the number of participants excluded for failing such a manipulation check (14% as opposed to 27%). Findings suggest emotion storyboards offer a method that can enhance participants’ accuracy when identifying social group memberships (e.g., race; socioeconomic status; gender) in studies on social judgments of emotion, relative to vignette manipulations. As with any method, researchers should be attentive when using the emotion storyboard that they are not relying on stereotypical depictions that reinforce essentialist thinking about social group members. One way the emotion storyboard method is able to do so is through its ability to control for features that are confounded with emotional expressions, such as gender and facial dominance cues [39].

Contrary to predictions, emotion storyboards and vignettes did not differ on immersion. Immersion ratings were around the mid-point of the scale for both emotion storyboards and vignettes, indicating that participants found the brief nature of each only moderately immersive. This finding reflects discussion [48] of the difficulties of fully immersing participants in vignettes without engaging all of people’s senses. Stronger effects of immersion and interest, however, might emerge in longer and more complex narratives. Indeed, research comparing longer graphic novel selections to text selections found graphic novels resulted in greater interest in learning more about the focal topic [49], and comparison of graphic novels to traditional novels found participants reported greater interest and enjoyment for the graphic novel than traditional novel [50].

Clarity explained effects of the emotion storyboard on comprehension inconsistently across studies. Future research should test other possible mechanisms that may explain the emotion storyboard’s enhancing effect on comprehension and comprehension response time. For instance, multimedia formats that fuse words and pictures can enhance learning and reduce cognitive load [51]. As others have discussed [51], research on the dual-code theory [52] suggests that people have two separate channels for processing verbal/auditory and visual/pictorial information, each of the channels has a limited capacity [53], and meaningful learning requires cognitive processing on several levels, for example through attention, organization, and integration [54]. One possibility is that emotion storyboards result in greater comprehension or faster response times on comprehension questions than vignettes, due to the multimodal nature of their design. Emotion storyboards could result in processing content through both channels, while, relative to emotion storyboards, vignettes could overload the verbal/auditory channel. In line with this possibility, second language learners had greater comprehension after reading graphic novels than text alone, irrespective of their pre-existing English language reading abilities or cognitive learning styles [49]. We did not find, however, that participants read the story more quickly in emotion storyboard format than vignette format. Such a finding might emerge, due to for instance reduced cognitive load, in a test with longer stories.

Another possibility is that the mutually-reinforcing combination of visual images and text in the emotion storyboard aids comprehension and comprehension response time. For instance, related pictures and text promoted greater attention and recall of health behaviors than text only [55]. Similarly, children who viewed a congruent picture rather than an incongruent picture while hearing a story narration had better comprehension of story details [56]. Eye-tracking data with these children revealed that they processed the congruent picture in a manner that maximally integrated narration and image. Additionally, the spatial organization of the emotion storyboard resembles a diagram and such a formation may enhance problem-solving through organization of information in spatially adjacent and logical locations [57]. The mutually-reinforcing nature of the emotion storyboard may also enhance participants’ confidence in their comprehension as evidenced by quicker response times to comprehension questions in emotion storyboard conditions, without sacrificing accuracy [58]. Future research could examine the deliberation process involved in responding to such questions using methods such as cursor-tracking [59].

It also remains possible, given comprehension and comprehension response times in both conditions were relatively high, that clarity may most robustly mediate the emotion storyboard’s effectiveness on such outcomes if comprehension questions were more difficult, if questions had free-recall rather than forced choice options, or if time was extended between reading the stories and answering comprehension questions. Additionally, power analyses were conducted for between-subjects effects rather than for mediation effects. Another possibility is that clarity and comprehension could be further enhanced in emotion storyboards relative to vignettes if the characters were depicted as more visually distinct from one another. In both studies, depictions of the characters’ clothing and physical features looked relatively similar. Future studies using emotion storyboards could pilot and use characters that have been rated as visually distinct from one another.

In vignettes, clarity was enhanced for participants who read about the Asian character as opposed to the White character. This unexpected finding could suggest that a feature of the vignettes, such as encountering a name that was potentially more novel to our predominately White participants (“Xiang” rather than “Andrew”), may have made participants especially attentive to the story and thereby affected perceptions of story clarity.

Findings suggest both emotion storyboards and vignettes are effective at conveying anger at a particular intensity and at conveying emotion as appropriate for a given situation. Although participants who read vignettes envisioned the anger of the main character as similarly appropriate in emotion type to those who read emotion storyboards, participants who read vignettes rated the character’s anger as less controlled and less appropriately intense than those who read emotion storyboards. These findings reveal when examining social judgments of emotion, subtle changes in nonverbal depictions can affect judgments. The unexpected interaction that emerged for the intensity of sadness manipulation check in the emotion storyboard conditions also highlights such effects. When judging the main character’s emotion in Study 1 when he confronted the rumor spreader, but not the lunch thief, participants who read the emotion storyboard made a more holistic judgment of that confrontation by including sadness they had seen the character express in a previous panel. Future research should meticulously examine visual and written cues about context and emotion that may be embedded in stimuli and may differentially affect such judgments relevant to emotion expression. These findings, in concert with greater clarity, greater accuracy of character race, and in some instances, enhanced comprehension and comprehension response time, for emotion storyboards than vignettes, suggest promise of the emotion storyboard to provide more emotional nuance through nonverbal depictions than vignettes may be able to feasibly include without sacrificing comprehension. For measures where effects of emotion storyboards and vignettes were not different, such as immersion, vignettes did not demonstrate an advantage over emotion storyboards. Thus, results suggest emotion storyboards are comparable to vignettes on a number of indicators and have advantages to vignettes on some indicators.

### Limitations and future directions

One limitation is that we only tested anger within the workplace context. Future directions should examine the effectiveness of emotion storyboards with other emotions, other contexts, other scenarios, and main characters with other social group memberships. Another limitation is that we tested some variables (e.g., scenario, race manipulation check) in only one study, which prevented us from testing the replication of their effects. Also, instead of a design comparing a “typical” narrative vignette and emotion storyboard, we could have created a vignette by verbally describing each of the illustrations in the emotion storyboard. Indeed, a future study could focus on specific written descriptions of the main character’s emotional expression (e.g., “scowling”, “arms crossed”) instead of descriptions of their emotion experience (e.g., “angrily”). Future research could examine cognitive processing constraints and other measures of relative effectiveness between emotion storyboards and vignettes as the level of written detail of the emotional expression is increased.

Additionally, we examined social interactions and emotional exchanges at only one level of complexity. Future research should compare the emotion storyboard and vignette methods with increasing complexity. To be sure, there may be elements of story complexity that might be more effectively conveyed in written vignette than in emotion storyboard. However, there are also elements of story complexity, such as time shifts or representation of multiple perspectives, that might be more rapidly and effectively conveyed through emotion storyboard than written vignette. Given potential cognitive processing constraints of providing thorough information about the context and other social actors [60], which may add both length and complexity to written text, it is possible the emotion storyboard method may be especially useful in instances in which story content is high in complexity. Indeed, guidelines for researchers using vignettes recommend minimizing vignette complexity as a design feature that should guide research practice [61]. One researcher suggested that vignettes should make three or fewer changes to the story of a vignette to prevent participant confusion [62]. Our findings of greater clarity and, although less consistent, findings of enhanced comprehension and comprehension response time for emotion storyboards than vignettes in these relatively simple social interactions, suggest that the emotion storyboard method may be even more effective than vignettes in depicting complex, multi-person emotion exchanges. If so, the emotion storyboard method could allow researchers the opportunity to examine questions that may have previously been avoided with written vignettes due to complexity constraints.

Another limitation of emotion storyboards is the requirement of a graphic artist for development. Although the cost of employing a graphic artist is likely less than the cost of producing high-quality videos [48], the cost of the method is higher than that of written vignette. However, once developed the illustrations can be easily customized and adapted to depict other contexts and emotional qualities using computer software without additional cost. Researchers interested in finding a graphic artist to develop emotion storyboards could advertise with their university or college art departments to find interested art students to work with. Alternatively, researchers could contract graphic artists for freelance work from online platforms.

A strength of emotion storyboards is that they enable researchers to selectively include and emphasize particular features of the social context. For instance, reactions of other characters to a main character expressing emotion can be easily included or excluded in the emotion storyboard according to the researcher’s goals. Specific content can be emphasized relative to other content through enlargement of a certain panel or through allocating multiple panels to a particular concept. In contrast, researchers using vignettes may have difficulty controlling which specific features of the social context participants attend to during the reading process. Future research is needed to examine how participants process content in emotion storyboards, using methods such as eye-tracking to connect basic-level processing to attention, comprehension, deliberation, and other relevant outcomes.

The emotion storyboard also provides researchers with strong experimental control of nonverbal elements in the social context. On the macro level, researchers can easily control specific nonverbal emotion information participants see and do not see, such as a character’s neutral expression prior to an emotion-relevant event. Across domains in social psychology, the emotion storyboard method offers researchers a way to easily convey and control complex social exchanges and story lines visually that may be cumbersome and confusing to convey via written text alone. On the micro level, the emotion storyboard method allows researchers to visually depict tone in emotion. Artistic techniques in graphic novels such as steam coming out of the ears of a character experiencing anger or sweat droplets depicted on a character experiencing nervousness can be used as metaphorical depictions to communicate emotion-relevant features, such as intensity of emotion. Although metaphorical and other descriptions of emotional tone can be similarly used in written form, a visual depiction may be a more efficient method than written vignette (as indicated, for example, by enhanced comprehension and comprehension response time) to convey particular concepts. In the emotion storyboards used in the present studies, sharing the rumor while covering one’s face, for instance, may have communicated additional felt emotion of the sharer, such as guilt. The act of covering one’s mouth with a hand may have also conveyed the manner in which the information was shared, for instance quietly. Future directions should establish and examine elements of emotional tone in visual depictions of social emotion.

Emotion storyboards may also offer a less intrusive way than other methods to examine how social group memberships affect emotion judgments. In vignettes it can be both difficult to represent various social group memberships without being too subtle (e.g., using names stereotypical of particular social groups) and without eliciting reactance from participants by being too explicit (e.g., “William, a Black man…”). Videos and photographs, unless digitally altered, are affected by confounds between gendered and racialized facial features and emotion expression [39]. By controlling for these features, emotion storyboards offer a more direct way to examine in future studies how stereotypes affect perception and evaluation of emotional behavior. Future directions could compare the effects of social group membership when manipulated through the emotion storyboard and vignette on judgments prone to stereotypes, such as perceptions of having an emotional disposition [38]. Future research could also compare the effects of controlled facial features in emotion storyboards to naturally occurring facial features in pictures or videos that affect judgments of emotion, such as affiliation and dominance perceptions [39]. Additionally, future research could use the emotion storyboard method to examine questions about cultural effects on social judgments of emotion, such as perceptions of emotion intensity [63, 64].

Additional future directions for the emotion storyboard method could include comparing the method’s effectiveness to other methods used to examine social judgments of emotion. Other such methods include videos, such as Movie for the Assessment of Social Cognition [65], and virtual reality techniques, such as Virtual Assessment of Mentalising Ability [66]. Further, the emotion storyboard method may have applicability beyond the study of social judgments of emotion. Future studies could examine the relative effectiveness of using emotion storyboards in other domains that commonly use vignettes, such as in tests of emotional intelligence and situational judgment tests used for personnel selection [e.g., 67, 68].

## Conclusions

Using a format inspired by graphic novels, the emotion storyboard method offers a useful way to examine social judgments of emotion. The emotion storyboard is comparable to the vignette method, with the advantage of enhancing clarity, accuracy of manipulation checks for social group memberships such as character race, and in some instances, comprehension and comprehension response time. The method enables researchers to visually convey, control, and emphasize elements of complex social exchanges. It further allows for visual inclusion of emotional nuance and emotional tone through nonverbal depictions. The method has implications for the study of social phenomena, such as stereotyping research.

## Supporting information

S1 Appendix Study 1 items for measures. (DOCX)

S2 Appendix Study 1 results. (DOCX)

S3 Appendix Study 2 items for measures. (DOCX)
