# Supplementary material for: The emotion storyboard: A method to examine social judgments of emotion
Source: PLoS One. 2021 Apr 2;16(4):e0249294. doi: 10.1371/journal.pone.0249294 (PMC8018665; doi:10.1371/journal.pone.0249294)
Supplement: S1 Appendix — (DOCX) [file pone.0249294.s001.docx]

**S1 Appendix. Study 1 items for measures.**

**Emotion type/intensity**

1. How angry was the main character when saying something to Paul?

2. How sad was the main character when saying something to Paul?

3. How surprised was the main character when saying something to Paul?

4. How afraid was the main character when saying something to Paul?

5. How disgusted was the main character when saying something to Paul?

**Comprehension**

1. Why was the main character angry?

a. A rumor was spread about him

b. He was blamed for something at work

c. Someone stole his lunch

d. His project was reassigned to another coworker

2. Who did the main character confront?

a. The person who started a rumor about him

b. The person who told him a rumor was going around about him

c. The person who told him someone was stealing his lunch

d. The person who stole his lunch

**Clarity**

1. How confusing was the story you read? (reverse-coded, R)

2. How clear was the story you read?

3. How easy was it to follow what was happening between the characters in the story?

4. How difficult was it to follow what emotions the characters in the story were feeling? (R)

**Immersion**

1. How interesting was the story you read?

2. How absorbed in the story were you?

3. How immersed in the story were you?

4. To what extent did you identify with any of the characters in the story?

**Emotional control (from Zawadzki, Warner, & Shields, 2013)**

1. How much self-control did the main character demonstrate?

2. How much were the main character’s feelings kept “in check”?

3. How much composure did the main character demonstrate?

**Appropriateness of emotional intensity (from Warner & Shields, 2009)**

1. The main character was too emotional. (R)

2. Most people would not have been so emotional at certain points as the main character was. (R)

3. I think that the main character had too much emotion for clear thinking. (R)

4. The emotions shown by the main character were too extreme. (R)

5. I think the main character was emotionally out of control. (R)
