# Supplementary material for: The emotion storyboard: A method to examine social judgments of emotion
Source: PLoS One. 2021 Apr 2;16(4):e0249294. doi: 10.1371/journal.pone.0249294 (PMC8018665; doi:10.1371/journal.pone.0249294)
Supplement: S2 Appendix — (DOCX) [file pone.0249294.s002.docx]

**S2 Appendix. Study 1 results.**

**Manipulations checks.**

***Main effects of format.*** For fear, there was no effect of format on perceptions of the main character’s intensity (*p* = .338, *η_p_^2^* = .01, 95% CI [0.00 to 0.04]; emotion storyboards *M* = 1.73, *SD* = 1.02; vignettes *M* = 1.88, *SD* = 1.21). For disgust, there was no effect of format on perceptions of the main character’s intensity (*p* = .335, *η_p_^2^* = .01, 95% CI [0.00 to 0.04]; emotion storyboards *M* = 4.61, *SD* = 1.52; vignettes *M* = 4.38, *SD* = 1.75). Surprise did not differ based on format (*p* = .307, *η_p_^2^* = .01, 95% CI [0.00 to 0.04]; emotion storyboards *M* = 3.34, *SD* = 1.74; vignettes *M* = 3.54, *SD* = 1.80). Sadness did not differ based on format (*p* = .767, *η_p_^2^* = .00, 95% CI [0.00 to 0.02]; emotion storyboards *M* = 2.89, *SD* = 1.63; vignettes *M* = 2.76, *SD* = 1.55).

***Main effects of scenario.*** Fear intensity did not differ based on scenario (*p* = .358, *η_p_^2^* = .00, 95% CI [0.00 to 0.04]). Disgust intensity did not differ based on scenario (*p* = .635, *η_p_^2^* = .00, 95% CI [0.00 to 0.03]). Perceptions of the main character’s surprise differed, (*F*(1, 190) = 11.63, *p*=.001, *η_p_^2^* = .06, 95% CI [0.01 to 0.13]), such that main characters in the rumor situation (*M* = 3.84, *SD* = 1.74) were rated as experiencing more surprise than those in the lunch stealing situation (*M* = 3.01, *SD* = 1.71). Perceptions of the main character’s sadness differed, (*F*(1, 190) = 10.76, *p* = .001, *η_p_^2^* = .05, 95% CI [0.01 to 0.13]), such that main characters in the rumor situation (*M* = 3.17, *SD* = 1.60) were rated as experiencing more sadness than those in the lunch stealing situation (*M* = 2.44, *SD* = 1.50).

***Interaction effects of format and scenario.*** For fear, there was no difference based on the interaction between format and scenario (*p* = .758, *η_p_^2^* = .00, 95% CI [0.00 to 0.02]). For disgust, there was no difference based on the interaction between format and scenario (*p* = .106, *η_p_^2^* = .01, 95% CI [0.00 to 0.06]). For surprise, there was no difference based on the interaction between format and scenario (*p* = .655, *η_p_^2^* = .00, 95% CI [0.00 to 0.03]).
