# Supplementary material for: The emotion storyboard: A method to examine social judgments of emotion
Source: PLoS One. 2021 Apr 2;16(4):e0249294. doi: 10.1371/journal.pone.0249294 (PMC8018665; doi:10.1371/journal.pone.0249294)
Supplement: S3 Appendix — (DOCX) [file pone.0249294.s003.docx]

**S3 Appendix. Study 2 items for measures.**

**Comprehension**

1. Why was the main character angry?

a. A rumor was spread about him

b. He was blamed for something at work

c. Someone stole his lunch

d. His project was reassigned to another coworker

2. How did the main character discover that it was Paul who spread a rumor about him?

a. The main character caught Paul in the act

b. A coworker said it was Paul

c. Multiple people told the main character it was Paul

3. How many characters were there in the story you read?

a. 2

b. 3

c. 4

d. 5

**Clarity** (Same as Study 1)

**Appropriateness of emotion type (from Warner & Shields, 2009)**

1. The emotions displayed by the main character were wrong. (R)

2. I would not have shown the types of emotions that the main character displayed. (R)

3. The main character's emotions were exactly the kinds that were called for.

4. I think the types of emotions that the main character felt were normal.

**Race manipulation check**

What do you think was the race/ethnicity of the main character who became angry?
